# 2D Transition Metal Dichalcogenides: Design, Modulation, and Challenges in Electrocatalysis

**DOI:** 10.1002/adma.201907818

**Published:** 2020-06-24

**Authors:** Qiang Fu, Jiecai Han, Xianjie Wang, Ping Xu, Tai Yao, Jun Zhong, Wenwu Zhong, Shengwei Liu, Tangling Gao, Zhihua Zhang, Lingling Xu, Bo Song

**Affiliations:** ^1^ School of Physics Harbin Institute of Technology Harbin 150001 China; ^2^ National Key Laboratory of Science and Technology on Advanced Composites in Special Environments Harbin Institute of Technology Harbin 150001 China; ^3^ School of Chemistry and Chemical Engineering Harbin Institute of Technology Harbin 150001 China; ^4^ Interdisciplinary Science Research Center Harbin Institute of Technology Harbin 150001 China; ^5^ Institute of Functional Nano and Soft Materials Laboratory (FUNSOM) Jiangsu Key Laboratory for Carbon‐Based Functional Materials and Devices Soochow University Suzhou 215123 China; ^6^ School of Advanced Study Taizhou University Taizhou 317000 China; ^7^ School of Environmental Science and Engineering Sun Yat‐sen University Guangzhou 510006 China; ^8^ Institute of Petrochemistry Heilongjiang Academy of Sciences Harbin 150040 China; ^9^ School of Materials Science and Engineering Dalian Jiaotong University Dalian 116028 China; ^10^ Key Laboratory for Photonic and Electronic Bandgap Materials Ministry of Education School of Physics and Electronic Engineering Harbin Normal University Harbin 150025 China

**Keywords:** electrocatalysis, hydrogen evolution reaction, performance modulation, synthesis methods, transition metal dichalcogenides

## Abstract

Hydrogen has been deemed as an ideal substitute fuel to fossil energy because of its renewability and the highest energy density among all chemical fuels. One of the most economical, ecofriendly, and high‐performance ways of hydrogen production is electrochemical water splitting. Recently, 2D transition metal dichalcogenides (also known as 2D TMDs) showed their utilization potentiality as cost‐effective hydrogen evolution reaction (HER) catalysts in water electrolysis. Herein, recent representative research efforts and systematic progress made in 2D TMDs are reviewed, and future opportunities and challenges are discussed. Furthermore, general methods of synthesizing 2D TMDs materials are introduced in detail and the advantages and disadvantages for some specific methods are provided. This explanation includes several important regulation strategies of creating more active sites, heteroatoms doping, phase engineering, construction of heterostructures, and synergistic modulation which are capable of optimizing the electrical conductivity, exposure to the catalytic active sites, and reaction energy barrier of the electrode material to boost the HER kinetics. In the last section, the current obstacles and future chances for the development of 2D TMDs electrocatalysts are proposed to provide insight into and valuable guidelines for fabricating effective HER electrocatalysts.

## Graphical abstract

Transition metal dichalcogenides (TMDs) are considered to be promising candidates over noble metal catalysts for electrochemical hydrogen production. The basic mechanism for the hydrogen evolution reaction (HER) is introduced, followed by a description of the different synthesis methods and modulation approaches to enhance the catalytic performance of TMD‐based catalysts toward the HER.

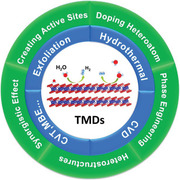

## 1 Introduction

Fast‐growing energy consumption has drawn great attention to the investigation of sustainable energy sources.^[^ ^1^, ^2^, ^3^, ^4^, ^5^, ^6^, ^7^ ^]^ Notably, hydrogen (H_2_), as one burgeoning promising energy carrier, owns a relatively high energy density (about 140 MJ kg^−1^) compared to other chemical fuels, and shows its ability to be the promising substitute for fossil energy.^[^ ^8^, ^9^, ^10^, ^11^, ^12^, ^13^, ^14^, ^15^ ^]^ In industry, steam reforming technique is the mainly used strategy to produce hydrogen, but this method may cause the undesirable environment pollution. In comparison, electrolysis of water will be a more eco‐friendly method to generate pure H_2_ (H_2_O (liquid) → H_2_ (gas) + 1/2 O_2_ (gas), Δ*G*_0_ = +273.2 kJ mol^−1^).^[^ ^16^, ^17^ ^]^ In order to reduce the electricity consumption and accelerate the reaction kinetics for HER process, electrocatalysts are commonly utilized. So far, platinum (Pt), iridium (Ir), and some other precious‐metal‐based compounds are the best electrocatalytic materials for HER; nonetheless, the scarcity and exorbitant price have restricted their large‐scale application.^[^ ^18^, ^19^, ^20^, ^21^, ^22^, ^23^, ^24^, ^25^ ^]^ Therefore, developing HER catalysts with cheap but effective materials, which shows similar property to Pt‐group metals (PGMs), has drawn significant attention. In the past decades, transition metals and their related compounds have been prepared and utilized as catalysts for water electrolysis, and some of them even shows the similar HER activity compered to PGM based catalyst.^[^ ^26^, ^27^, ^28^, ^29^, ^30^, ^31^, ^32^, ^33^, ^34^, ^35^, ^36^, ^37^ ^]^ In particular, 2D TMDs nanosheets (NSs), for example, molybdenum diselenide (MoSe_2_),^[^ ^38^, ^39^ ^]^ tungsten diselenide (WSe_2_),^[^ ^40^, ^41^ ^]^ tungsten disulfide (WS_2_),^[^ ^42^, ^43^, ^44^ ^]^ and molybdenum disulfide (MoS_2_),^[^ ^45^, ^46^, ^47^ ^]^ are increasingly attracting attention owing to their excellent catalytic property.

Compared to other earth‐abundant catalysts, the 2D TMDs show some unique characteristics: 1) the 2D layered structure enables the TMDs‐based electrocatalysts to offer an increased specific surface area with plentiful reaction sites for HER; 2) the measured in‐plane resistivity of 2D TMDs NSs is smaller than the resistivity through the basal planes, which would lead the electrons to transport more easily along the basal plane and reach the catalytic sites at the edges;^[^ ^48^, ^49^ ^]^ and 3) the d orbitals of different transition metals are various, which results in versatile electronic structures of TMDs, which eventually leads to distinct catalytic behavior. Moreover, as one of the earliest studied electrocatalysts, MoS_2_ and other TMDs catalysts exhibit significant boost in their catalytic activity over the last decade. Further, their HER catalytic mechanisms are deeply investigated and comprehensively understood, which provide valuable insight into the development of new HER catalysts.

Herein, some of the recent important progresses of TMDs‐based HER electrocatalysts will be summarized. Owing to the rapid development in this booming field, we may not be able to cover the entire body of explorations related to TMDs electrocatalysts. Instead, fundamental understanding of preparation and modulation strategies is highlighted and reviewed herein, which aims to provide the readers with a clear perception of the development methodologies of 2D TMDs. After the background introduction, the universal methodologies for the preparation of 2D TMDs electrocatalysts, e.g., exfoliating the bulk materials, hydrothermal method, and chemical vapor deposition (CVD) are outlined. Next, the advantages and potential applications of different methodologies are also presented. Subsequently, this review mainly provides in‐depth insight into the strategies promoting the HER activity of TMDs‐based materials, including increasing active sites number, heteroatoms doping, phase engineering, constructing heterostructures, and synergistic modulation. Finally, challenges and opportunities of 2D TMDs electrocatalysts for the hydrogen production technologies in future are proposed.

## 2 Electrochemical HER

### 2.1 Mechanism of HER

Two half reactions happened on the different electrodes make up the integrated electrocatalytic water splitting process: the HER process and the oxygen evolution reaction (OER) process

(1) $${\text{H}}_{2}\text{O}\to {\text{H}}_{2}+1/2\;{\text{O}}_{2}$$

In fact, there exist two different reaction mechanisms for HER in the electrolyte:

Volmer–Tafel (V–T) mechanism: In this mechanism, a transferred electron first combines with a proton and is adsorbed by the catalyst, forming the adsorbed hydrogen atom (${\text{H}}_{\text{ads}}^{\text{*}}$) at the active site of the catalyst (which is also called Volmer or discharge reaction step); subsequently two ${\text{H}}_{\text{ads}}^{\text{*}}$ combine and generate H_2_ gas (the Tafel step). This mechanism can be described as follows (the symbol “*” represents the hydrogen adsorption site)

(2) $$\text{Volmer}\;\text{step}:{\text{H}}^{+}(\text{aq})+{\text{e}}^{-}+*\to {\text{H}}_{\text{ads}}^{*}$$

(3) $$\text{Tafel}\;\text{step}:2{\text{H}}_{\text{ads}}^{*}\to {\text{H}}_{2}(\text{g})+2*$$

Volmer–Heyrovsky (V–H) mechanism: Different from the V–T mechanism, after the Volmer reaction, the ${\text{H}}_{\text{ads}}^{\text{*}}$ tends to combine with a H^+^ in electrolyte and another transferred electron from external circuit and subsequently H_2_ gas is formed (the Heyrovsky step). In this circumstance, the HER process goes through the following route

(4) $$\text{Volmer}\;\text{step}:{\text{H}}^{+}\text{(aq)}+{\text{e}}^{-}+*\to {\text{H}}_{\text{ads}}^{*}$$

(5) $$\text{Heyrovsky}\;\text{step}:{\text{H}}_{\text{ads}}^{*}+{\text{H}}^{\text{+}}(\text{aq})+{\text{e}}^{\text{–}}\to {\text{H}}_{2}(\text{g})+*$$

For both of the mechanisms mentioned above, the rate‐determining step (RDS) are usually evaluated on the basis of the Tafel slope, which is further expounded in Section 2.2.2.^[^ ^50^, ^51^ ^]^

**Figure 1:**
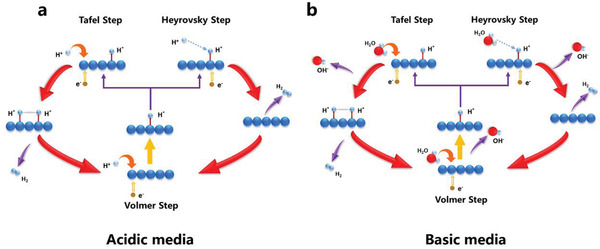
a,b) Different pathways for HER: a) in acidic media, and b) in basic media.

Moreover, the HER process is also a pH‐dependent reaction. The processes in acidic and basic solutions show some differences, mainly in the Volmer step. In acidic media, the H^+^ is abundant, which indicates that the hydrogen adsorption sites can obtain the protons easily via Volmer reaction. Further, the ${\text{H}}_{\text{ads}}^{*}$ goes through Tafel or Heyrovsky step to generate H_2_ gas (shown in **Figure 1**a). However, when the basic medium is used as the electrolyte (alkaline or neutral), the HER process is restricted because of the lack of proton, and the water molecules (H_2_O) have to act as the proton donator for the subsequent reactions, which will be dissociated into H^+^ and OH^−^ at the first step. This is the origin for the sluggish reaction rate for HER in basic media (as shown in Figure 1b).^[^ ^52^, ^53^ ^]^

### 2.2 Primary Parameters for Evaluating Catalytic Activity of the Catalysts

Notably, some primary parameters are usually used to evaluate the catalytic performance for a certain material. It is extremely important to deeply understand these parameters for designing an efficient catalyst. In the following sections, some widely recognized parameters are introduced.

#### 2.2.1 Gibbs Free Energy and Overpotential (η)

**Figure 2:**
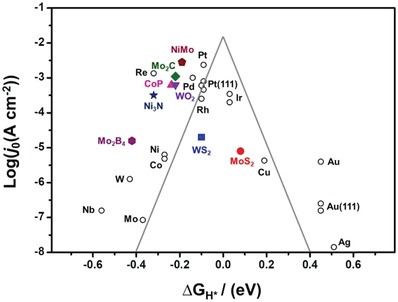
Volcano plot that shows the relationship of exchange current density of various materials and their corresponding $\Delta {\text{G}}_{{\text{H}}^{\text{*}}}$. Reproduced with permission.^[^ ^53^ ^]^ Copyright 2019, Elsevier.

Regardless of different reaction mechanisms mentioned above (the V–T mechanism or V–H mechanism), the ${\text{H}}_{\text{ads}}^{\text{*}}$, which is intermediately generated during the mutual Volmer reaction, is always required for following HER step. Therefore, the Gibbs free energy for adsorbing hydrogen atom ($\Delta {\text{G}}_{{\text{H}}^{\text{*}}}$) is a commonly used parameter to describe the HER performance for the catalysts.^[^ ^54^, ^55^, ^56^, ^57^ ^]^ According to the Sabatier plot (also known as the Volcano plot, **Figure** **2**),^[^ ^53^ ^]^ the free energy for adsorption of reactants and reaction intermediates should be neither too low nor too high ($\Delta {\text{G}}_{{\text{H}}^{\text{*}}}$ ≈ 0, i.e., is the idealist value).^[^ ^50^, ^54^, ^58^ ^]^ PGMs usually demonstrate nearly zero $\Delta {\text{G}}_{{\text{H}}^{\text{*}}}$ and highest exchange current density, which results in the best catalytic performance toward HER. Metals on the left of the Pt group ($\Delta {\text{G}}_{{\text{H}}^{\text{*}}}$ < 0) bind strongly to hydrogen, making the first Volmer step relatively easy; nonetheless, it results in a difficult hydrogen desorption step, thus affecting the following Tafel or Heyrovsky steps and eventually poisoning the catalyst surface. In contrast, if the metals are located at the right side of Pt group ($\Delta {\text{G}}_{{\text{H}}^{\text{*}}}$ > 0), a relatively weak binding ability toward hydrogen molecule is observed; therefore, to start the Volmer step, more energy is needed, which limits the overall catalytic performance. Therefore, $\Delta {\text{G}}_{{\text{H}}^{\text{*}}}$ is an important evaluation criterion for screening the potential electrocatalysts for HER. MoS_2_, as a typical TMDs, has a modest value of $\Delta {\text{G}}_{{\text{H}}^{\text{*}}}$, which is close to the PGMs, and indicates the potential as a highly efficient HER catalyst (Figure 2).^[^ ^53^ ^]^

The value of $\Delta {\text{G}}_{{\text{H}}^{\text{*}}}$ is either higher or lower than zero practically, which indicates that a thermodynamic η is needed for driving the HER process. Three possible sources are responsible for the increase of η: 1) the activation potential, which depends on the intrinsic property of the catalytic material; 2) the concentration potential, which is influenced by the uniformity coefficient of the electrolyte; and 3) the resistance potential, induced by the electrochemical interface. The HER performance of the catalyst can be roughly evaluated by the value of the applied voltage which is needed to reach a certain current density. In this case, two significant points should be focused on: one is the onset potential and the other is η. The former represents potential value when linear sweep voltammetry (LSV) curve shows remarkable bending.^[^ ^59^, ^60^ ^]^ The latter is a criterion for the efficiency of photo‐electrochemical (PEC) water splitting system in commercially equivalent and it is also a popular parameter of ranking electrocatalysts for water‐splitting reactions (usually recorded when the current density reaches 10 mA cm^−2^).^[^ ^53^, ^61^, ^62^ ^]^ Obviously, an excellent electrocatalyst should have the ability to generate H_2_ gas at a relatively low η. Given the origins of the η mentioned above, three corresponding methods, including selection of an efficient catalyst, stirring the solution while testing, and conducting Ohmic drop compensation for *iR* loss should be considered to minimize the η.^[^ ^63^ ^]^

#### 2.2.2 The Tafel slope and Exchange Current Density (*j*_0_)

As a frequently used parameter, the Tafel slope is used to uncover correlation of catalytic current density (*j*) and η which can be obtained from the Butler–Volmer kinetic model^[^ ^64^, ^65^ ^]^

(6) $$\left|\eta \right|\text{=}\frac{\text{2}.\text{3}RT}{\alpha nF}\;\text{log}\frac{j}{j_{\text{0}}}$$

In this equation, *R* and F are two constants, which represent ideal gas constant (8.314 J mol^−1^ C^−1^) and Faraday constant (96 485 C mol^−1^), respectively. *T*, α, and *n* represent Kelvin temperature, electrochemical transfer coefficient, and electrons that transferred, respectively. *j* and *j*_0_ represent catalytic and exchange current densities, respectively.

Tafel slope *b* (mV dec^−1^) could be written as

(7) $$b\;=\frac{\text{2}.\text{3}RT}{\alpha nF}$$

Notably, the value of *b* can be acquired by fitting of the Tafel plot within the linear portion. This value can be further used to deduce the catalytic mechanism occurring at the electrode.^[^ ^66^ ^]^ At room temperature, the Tafel slops for Tafel, Heyrovsky and Volmer steps were calculated to be about 30, 40, and 120 mV dec^−1^, respectively.^[^ ^67^ ^]^ The calculated Tafel slope indicate that, if the *b* is more than 120 mV dec^−1^, the RDS is the Volmer reaction, and the hydrogen atoms is too sluggish to be adsorbed on the catalysts surface. If the *b* is between 40–120 mV dec^−1^, Heyrovsky reaction becomes the RDS, and the hydrogen atom can get adsorbed on the catalyst surface more easily, but will be desorbed slowly. However, if the value of *b* turns out to be about 30 mV dec^−1^, Tafel reaction will be regarded as the RDS. This situation is usually observed from the Pt/C catalyst. Therefore, by referring to the Tafel slope, main HER pathway for a certain catalyst can be roughly inferred.

*j*_0_ is another descriptor to evaluate the ability of charge transfer from the electrode to the electrolyte, which is also a vital parameter to estimate the HER activity of the electrocatalysts. Larger value of *j*_0_ indicates a higher intrinsic activity under equilibrium conditions for the electrode material. In general, extrapolating the Tafel plots along its linear part to the point when the η becomes zero, the exchange current density is obtained.^[^ ^68^, ^69^ ^]^

#### 2.2.3 Other Useful Descriptors for HER

Besides the useful parameters mentioned above, other indicators such as electrochemically active surface area (ECSA), turnover frequency (TOF), stability, and faradaic efficiency (FE) are also important descriptors to assess the catalytic activity for a material. In particular, the ECSA is important for the evaluation of the HER or OER performance, because its value reflects the approximate information about the quantity of active sites on the catalysts’ surface. Although maybe not all sites could show catalytic activity in the reaction, the ECSA can still serves as a reference for evaluating the performance.^[^ ^70^, ^71^, ^72^ ^]^ For the comprehensive investigation of a catalyst’s activity (in particular, the well‐defined molecular catalysts), the efficiency of each active site should be investigated. The TOF is used to evaluate the quantity of the generated H_2_ molecules per second at one single active site. However, in the actual situation, the calculated TOF is usually imprecise because of the difficulty to confirm the total active sites, and thus the TOF calculation often provides just a rough estimate.^[^ ^46^, ^73^, ^74^ ^]^ Furthermore, long‐term stability is usually regarded as an important parameter to evaluate the potential for practical application. The stability can be tested by cyclic voltammetry (CV),^[^ ^75^ ^]^ chronoamperometric analyses,^[^ ^76^ ^]^ or chronopotentiometric analyses.^[^ ^77^ ^]^ A catalyst with excellent durability must demonstrate negligible variation during the CV cycles (usually 1000 cycles or more) and the LSV curve before and after long‐term (at least 10 h or longer) measurement should also be as close as possible. FE is used to define the ratio of electrons that participates during the HER process versus the overall electrons transferred from the external circuit. Moreover, FE is generally less than 100% due to the occurrence of the concomitant side reactions.^[^ ^78^, ^79^ ^]^

Noteworthy, a single parameter may not be able to reflect the catalytic performance systemically and objectively. For example, the value of η could be influenced by both the catalyst loading and the ECSA of the specific electrode. Therefore, if we only consider the value of η and ignore other related parameters, the description of catalytic performance may be imprecise. As a result, to clearly understand the catalytic mechanism and the HER performance, various descriptors should be comprehensively applied.

## 3 Preparation of 2D TMDs‐Based Electrocatalysts

The excellent catalytic performance of TMDs‐based electrocatalysts largely relies on its layered structure, and thus the synthesis of layer‐controllable TMDs materials with large‐area uniformity is indispensable for their broad range of practical applications. Undeniably, significant achievements have been made to prepare high‐quality monolayer TMDs NSs. This section summarizes commonly used methodologies that are used to synthesize of 2D TMDs materials, consisting of the top to down approach by exfoliating the bulk counterparts and bottom to up approach such as hydrothermal (solvothermal) method and vapor‐phase deposition route.

### 3.1 Exfoliation from the Bulk Materials

The unexfoliated TMDs, such as MoS_2_,^[^ ^20^ ^]^ MoSe_2_,^[^ ^27^, ^80^ ^]^ and WS_2_,^[^ ^33^ ^]^ are semiconducting 2H phase (trigonal prismatic) materials, wherein the most catalytic active sites remain unexposed and the poor conductivity largely limits their catalytic activities. By exfoliating the bulk TMDs, layered TMDs NSs with larger surface area and abundant active sites can be obtained.^[^ ^81^, ^82^ ^]^ A direct method to obtain single‐layer TMDs NSs is mechanical exfoliation, whose synthetic process is similar to the synthesis of 2D graphene.^[^ ^83^ ^]^ Nevertheless, yield rate of mono‐ or few‐layer TMDs NSs with this technique is too low to satisfy the application in practical electrocatalysis, but more suitable for some mechanism investigations or the preparation of devices. To meet the needs of practical catalytic use, lithium insertion is proposed to be an efficient strategy to obtain layered TMDs materials in large scale. Lithium insertion process will not only reduce the layer number of bulk TMDs, but also be accompanied by some crystal structure chances, such as the phase transform in TMDs (usually from inert 2H phase to active 1T or 1T′ phase), thus further improving their catalytic activity toward HER. Three lithiation methods are commonly used to exfoliate the bulk TMDs materials.

**Figure 3:**
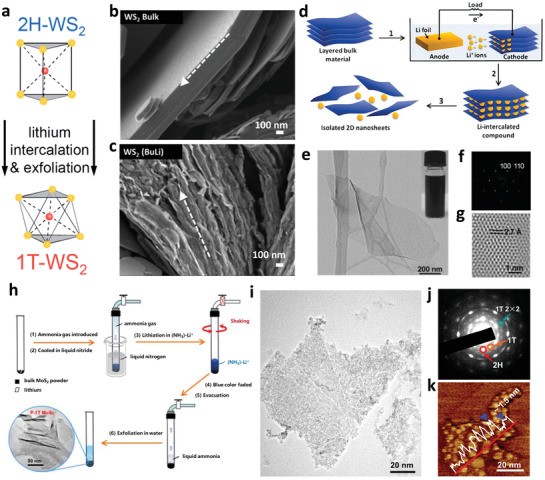
Three commonly used lithium‐insertion methods to obtain layered TMDs materials NSs. a) Exfoliating the bulk WS_2_ with organolithium compounds to produce 1T WS_2_ NSs. Reproduced with permission.^[^ ^33^ ^]^ Copyright 2014, Royal Society of Chemistry. b) The SEM images for the bulk WS_2_ and c) after the BuLi treatment with subsequent exfoliation in water. b,c) Reproduced with permission.^[^ ^89^ ^]^ Copyright 2014, American Chemical Society. d) Fabrication of 2D NSs from the layered bulk material with electrochemical lithiation process. e) TEM image of an exfoliated MoS_2_ NS. Digital photograph of the MoS_2_ NSs solution is demonstrated as the inset image. f) Selected‐area electron diffraction (SAED) of monolayer MoS_2_ NS. g) HRTEM image for the exfoliated monolayer MoS_2_ NS. d–g) Reproduced with permission.^[^ ^90^ ^]^ Copyright 2011, Wiley‐VCH. h) A diagrammatic drawing of LAAL processes. i) Morphology of mesoporous 1T MoS_2_ NSs. j) The corresponding SAED pattern and k) atomic force microscopy (AFM) image of exfoliated MoS_2_ NSs. h–k) Reproduced with permission.^[^ ^20^ ^]^ Copyright 2016, American Chemical Society.

The first one is the chemical exfoliation process with organolithium compounds such as butyllithium (BuLi),^[^ ^84^, ^85^, ^86^, ^87^, ^88^ ^]^ methyllithium (MeLi),^[^ ^89^ ^]^ or lithium borohydride (LiBH_4_)^[^ ^87^ ^]^ (**Figure** **3**a–c). In this method, bulk TMDs powder would be soaked into the solution, containing the lithium sources and corresponding organic solvent (such as hexanes), and continuous ultrasonication (usually more than 2 days) would be synchronously applied to make the exfoliation more efficient. Nevertheless, this method is time consuming, and the exfoliating effect was sometimes unsatisfying (Figure 3c). So, the high yield of monolayer NSs and the controllability over the lithium insertion process are still challenging.

In order to overcome the abovementioned limitations, another high‐yield electrochemical lithium insertion method was then proposed to fabricate 2D single‐layer nanomaterials, by a controllable lithiation reaction, which shows certain advantages over the former one.^[^ ^90^, ^91^, ^92^ ^]^ The major difference is that the lithium insertion was conducted in a lithium battery test cell (Figure 3d). Bulk TMDs at cathode of a cell was inserted by lithium from the anode during the discharge process and gradually exfoliated to layered NSs. After washing, sonication, and centrifugation, numerous single‐ or few‐layer NSs were obtained (Figure 3e–g).Yield via this electrochemical lithium insertion is higher compared to that via the chemical exfoliation (usually 10–20%),^[^ ^93^ ^]^ which could reach over 90% for MoS_2_,^[^ ^90^ ^]^ TaS_2_,^[^ ^94^ ^]^ and TiS_2_.^[^ ^94^ ^]^ However, this method still suffers from the drawbacks such as complicated process, which needs the assembly of battery cells. At the same time, additional additives that are usually used during the electrode fabrication process may introduce impurities into the final products.^[^ ^49^ ^]^

Very recently, our team proposed a unique liquid ammonia‐assisted lithiation (LAAL) technique for the exfoliation of bulk TMDs and it turned out to be an efficient way to obtain ultrathin 2D NSs.^[^ ^20^, ^95^ ^]^ To conduct the LAAL method (Figure 3h), lithium metal is first placed into a quartz tube under the argon (Ar) protection. Then, the tube is evacuated and dipped in a liquid nitrogen bath. At the same time, highly pure ammonia gas is introduced, which gradually condenses into liquid state. When the powder is immersed into liquid ammonia, the lithiation reaction begins immediately, and the color of the liquid also gradually changes (from blue color into colorless), which acts as the indicator for monitoring the reaction progress. When the “blue color” completely fades away, ammonia gas is carefully removed from the tube by evaporation. Further, by adding water into the lithium intercalated sample, the ultrathin 2D NSs are obtained (Figure 3i–k).

Compared with the other two methods mentioned above, the LAAL pathway demonstrated three obvious advantages: 1) this method will take a shorter time, usually within 1 h. And with the distinct change of color, the reaction process could be intuitively judged without using other indicator; 2) 1T phase single‐layer or few‐layer TMDs NSs could be easily obtained with a high yield (≈82%); 3) the drastic lithiation process will induce the formation of plentiful sulfur‐vacancies (S‐vacancies) and more edges, which will further enhance the electrochemical activity of exfoliated TMDs NSs. What should be noted is that, because of the violent reaction between water and metal lithium, as well as the application of liquid ammonia, all the operating steps must be carefully conducted to ensure the safety.

Briefly, thinning the layer of TMDs catalysts can be achieved by exfoliation the bulk materials, and various strategies have been proved to be efficient, especially the lithiation process. But due to the complex reaction process in liquid media, it is unable to obtain the TMDs NSs with expected properties, such as regular morphologies and controllable layer number, which makes it difficult to investigate the mechanism in electrocatalysis.

### 3.2 Hydro/Solvothermal Method

**Figure 4:**
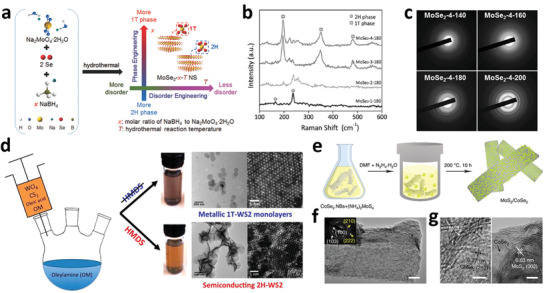
a) The influence of disorder engineering and phase engineering on MoSe_2_ NSs caused by adjusting the reductant ratio (*x*) and preparation temperature (*T*) with hydrothermal technique. b) Raman spectrum for different MoSe_2_ NSs sample synthesized with various ratio of NaBH_4_. The ratio of 1T phase MoSe_2_ gradually increases with the addition of NaBH_4_. c) SAED patterns of different MoSe_2_ samples synthesized with temperatures and showing better crystallinity at higher hydrothermal reaction temperatures. a–c) Reproduced with permission.^[^ ^27^ ^]^ Copyright 2017, Wiley‐VCH. d) Solvothermal synthesize process diagram and the corresponding TEM and HAADF‐HRSTEM images of WS_2_ NSs with different phases. Reproduced with permission. ^[^ ^101^ ^]^ Copyright 2015, American Chemical Society. e) Synthetic route of two‐component MoS_2_/CoSe_2_ hybrid catalyst. f) TEM and SAED images of MoS_2_/CoSe_2_ hybrid (scale bar, 50 nm). g) HRTEM result of the MoS_2_/CoSe_2_ hybrid. e–g) Reproduced under the terms of the CC‐BY Creative Commons Attribution 4.0 International License (http://creativecommons.org/licenses/by/4.0/).^[^ ^105^ ^]^ Copyright 2015, Springer Nature.

Hydro/solvothermal is a low‐cost and convenient way to synthesize TMDs‐based nanomaterials in large‐scale. By adjusting the temperature, reaction time, type of metal precursors, surfactants, and other experimental parameters, various samples with different morphologies, phases, or crystallinity can be obtained, which makes the hydro/solvothermal an ideal method to prepare nanostructured materials.^[^ ^96^, ^97^, ^98^ ^]^ Recently, MoSe_2_ NSs prepared by hydrothermal reaction was found to be an efficient HER catalyst. By tuning the reaction temperature, together with the ratio of NaMoO_4_·2H_2_O and Se precursors to the reductant (NaBH_4_), the products would show different crystal phases and disorder degree (**Figure 4**a).^[^ ^27^ ^]^ It showed that higher content of NaBH_4_ would increase the ratio of 1T MoSe_2_, which showed better HER activity (Figure 4b). On the other hand, lower reaction temperature would bring in more active sites, but go against the formation of 1T phase. By precisely optimizing the disorder degree and ratio of 1T phase, the MoSe_2_ NSs with the optimal HER catalytic activity were obtained. Hydrothermal reaction was a commonly used strategy to prepare nanostructured materials, but a shortcoming is that the products could be easily oxidized during the process, either from the atmosphere or from the solution, which may influence the purity of the produced materials. To avoid the possible oxidation, the solvothermal reaction is also applied for synthesizing TMDs‐based nanomaterials.^[^ ^99^, ^100^ ^]^ Another advantage for solvothermal reaction lies in its selectivity of the final products. For instance, by selectively adding hexamethyl disilazane (HMDS), 2H WS_2_ would be obtained. Otherwise, 1T WS_2_ would be finally produced (Figure 4d).^[^ ^101^ ^]^

Generally speaking, due to relatively low preparation temperature, the crystallinity of the as‐prepared materials is usually inferior to that prepared under high temperature such as solid‐state reaction. But this will also make the products with more active sites. And also, the plentiful combining forms of reactants will benefit for constructing heterostructured nanomaterials, such as MoS_2_/CuS,^[^ ^102^ ^]^ MoS_2_/CdS,^[^ ^103^ ^]^ MoS_2_–graphene,^[^ ^104^ ^]^ and MoS_2_/CoSe_2_^[^ ^105^ ^]^ with abundant interfaces and suitable level structures, which will benefit their electrocatalytic activity (Figure 4e–g).

### 3.3 CVD Method

**Figure 5:**
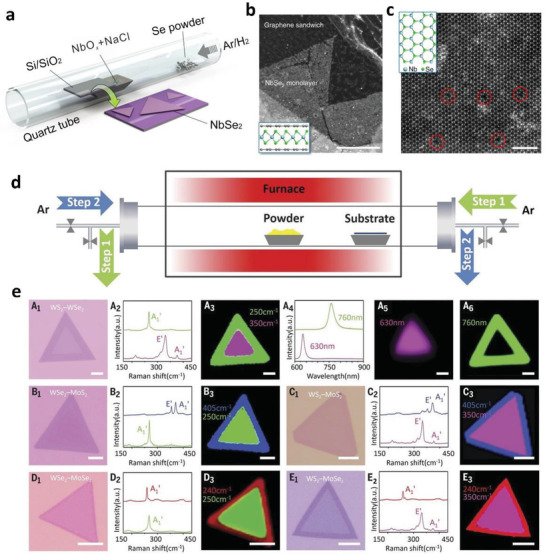
a) A typical CVD setup used to fabricate NbSe_2_ nanosheets. b) A low‐magnification annular dark‐field STEM (ADF‐STEM) image of single‐layer NbSe_2_ NSs encapsulated by the graphene sandwich. c) Atomic resolution ADF‐STEM of NbSe_2_ demonstrating the hexagonal crystal structure. a–c) Reproduced under the terms of the CC‐BY Creative Commons Attribution 4.0 International License (http://creativecommons.org/licenses/by/4.0/).^[^ ^106^ ^]^ Copyright 2017, The Authors, published by Springer Nature. d) Modified CVD system with reversible Ar flow for the epitaxial growth of the lateral TMDs heterostructures. e) Optical microscopy images for the grown various 2D NSs with lateral heterostructures using the setup and their corresponding characterizations. d,e) Reproduced with permission.^[^ ^119^ ^]^ Copyright 2017, The Authors, published by American Association for the Advancement of Science.

CVD shows great promise as an efficient method to obtain high‐quality, low‐defect, and layer controllable TMDs nanostructures or thin films on various substrates.^[^ ^106^, ^107^, ^108^, ^109^, ^110^, ^111^ ^]^ One of the advances for vapor‐phase deposition involves the adjustment of precursors ratio, i.e., the component of the final products can be facilely controlled and well contacted interfaces between nanostructures and substrates are favorable for charge migration. At the same time, high‐quality catalysts obtained via CVD method also provides a simple but pure platform to investigate the HER mechanism. In a typical CVD synthesis process, the metal precursors, such as metal films,^[^ ^112^, ^113^ ^]^ metal oxides,^[^ ^114^, ^115^ ^]^ metal halides,^[^ ^116^, ^117^ ^]^ or metalorganics,^[^ ^118^ ^]^ are placed in the furnace at center heating zone center, while S, Se, or Te powder is placed upstream relative to the flow direction of the carrier gas (usually Ar or N_2_ mixed with a certain concentration of H_2_). When the temperature increases, the S, Se, or Te vapor will flow downstream, which converts the metal precursor into the corresponding TMDs in the high temperature zone (**Figure** **5**a–c).^[^ ^106^ ^]^ A recent research demonstrated that 2D monolayer heterostructures could be obtained by switching the flowing direction of carrier gas.^[^ ^119^ ^]^ Usually, the lateral heterostructures with two different TMDs monolayer are delicate, and might be hard to survive in the multistep growth. To address this issue, Zhang et al. proposed a novel step‐by‐step synthesis strategy by reversing the gas flowing direction for growing various in‐plane 2D TMDs nano heterostructures. In order to conduct this strategy, a monolayer TMDs NS was first grown on the substrate with CVD method. For the sequential process, formerly grown monolayer was placed at the downstream of Ar flow, and Ar flowed from the reversed direction during the temperature swing, which cooled the existing monolayer TMDs materials and prevented the occurrence of undesirable thermal degradation. Meantime, the reverse Ar flow also restrained the uncontrolled nucleation before the sequential growth step (Figure 5d). This effective CVD technique could be used to grow a variety of in‐plane 2D lateral heterostructures (i.e., WS_2_–WSe_2_, WSe_2_–MoS_2_, and so on, see Figure 5d), and multi‐heterojunctions (i.e., WS_2_–WSe_2_–MoS_2_, WS_2_–MoSe_2_–WSe_2_).

To sum it up, the preparation of high‐quality TMDs NSs or heterostructures by CVD technique requires careful modulation of some important parameters. One is the synthetic temperature. Generally, high temperature will result in better crystallinity, but the nanostructure may be unstable under excessive temperatures. Thus, the temperature is the foremost parameter that should be carefully chosen. Another is the distance between the chalcogen and metal source. If the CVD set up is consisted with two separate heat sources, the distance would be inalterable. Then, the flow of carrier gas (usually Ar or N_2_) should be tuned to an appropriate value so that the TMDs NSs could be deposited on the substrates. However, if there is only one heating source, the temperature gradients in the tube should be calibrated in advance to determine the proper position for reactants. In this instance, both the distance and flow of carrier gas must be carefully adjusted together. Many works only demonstrate the schematic diagram of CVD apparatus when describing the experimental procedures, but ignore the exact distance between the reactants and the substrates (or the real reaction temperature), which would sometimes make it difficult to repeat these experimental results, due to the minute differences of CVD set up. Herein, it is suggested that the authors should provide all the detailed parameters to ensure the audiences could reproduce the experimental results in their own lab.

### 3.4 Other Methods of Synthesis

Besides the methods mentioned above, some other synthesis pathways have also been used for fabricating TMDs materials. For example, thermal annealing is a straightforward pathway to prepare TMDs‐based materials.^[^ ^120^, ^121^ ^]^ However, it is difficult to obtain TMDs with well‐controlled morphology and layer‐number; moreover, the high temperature may often lead to the material aggregation which limits the performance toward HER. Even so, some research attentions had been paid to this method for obtaining electrocatalysts with decent catalytic activity toward HER.^[^ ^122^, ^123^ ^]^ To obtain high‐quality bulk single crystal, chemical vapor transport (CVT) is usually utilized. In this technique, raw materials react with the transport agents (usually the pure halogen elements or their compounds) at the hot zone, and then are transported to the cold zone, where the growth of single crystals is observed. The growth temperature, transport agent, and reaction time are the main parameters that should be carefully tuned for obtaining the high‐quality TMDs single crystal.^[^ ^124^, ^125^, ^126^ ^]^ Molecular beam epitaxy (MBE) is another effective method to prepare high‐quality films.^[^ ^127^, ^128^ ^]^ Owing to the high‐purity of the as‐grown films, these samples are also suitable for investigating the origin of the catalytic activities in TMDs catalysts.^[^ ^129^, ^130^ ^]^

## 4 Strategies to Promote the Catalytic Performance of 2D TMDs

In the past few years, 2D TMDs electrocatalysts, such as MoS_2_,^[^ ^131^, ^132^ ^]^ MoSe_2_,^[^ ^133^, ^134^, ^135^ ^]^ WS_2_,^[^ ^43^ ^]^ niobium disulfide (NbS_2_),^[^ ^136^, ^137^ ^]^ etc., are proved to be promising candidates to meet the disadvantages of noble metals or related compounds. To maximize the utilization of the unique properties of 2D TMDs, some novel strategies have been proposed to further enhance the HER performance. In general, the strategies can be classified into five categories, including: 1) creating more active sites; 2) heteroatoms doping; 3) phase engineering; 4) construction of heterostructures; and 5) synergistic modulation. In this sections, recent achievements for improving the catalytic activity with these specific tactics are summarized.

### 4.1 Creating More Active Sites

**Figure 6:**
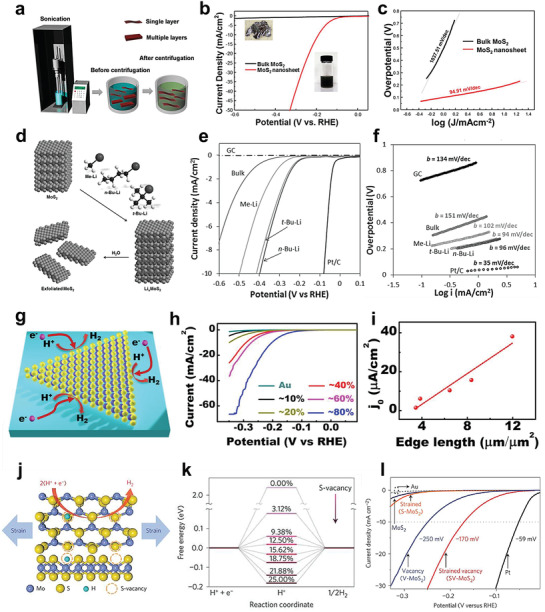
a) Diagram of liquid exfoliation method to synthesize TMDs NSs. b) LSV curves of the exfoliated and bulk MoS_2_ material and c) their corresponding Tafel slopes. a–c) Reproduced with permission.^[^ ^141^ ^]^ Copyright 2016, American Chemical Society. d) The exfoliating process of bulk MoS_2_ with different kinds of organolithium compounds. e,f) The electrochemical results of the MoS_2_ NSs exfoliated with different organolithium compounds. d–f) Reproduced with permission.^[^ ^142^ ^]^ Copyright 2014, Wiley‐VCH. g) MoS_2_ grown on Au substrate. h) Coverage‐dependent LSV curves. i) Statistical relationship between the edge lengths of MoS_2_ and the exchange current density. g–i) Reproduced with permission.^[^ ^114^ ^]^ Copyright 2014, American Chemical Society. j) Top and side views of S‐vacancies incorporated MoS_2_. k) $\Delta {\text{G}}_{{\text{H}}^{\text{*}}}$ of MoS_2_ with different S‐vacancies ratio. l) HER performance comparation of different MoS_2_ samples with various amounts of strain and S vacancies. The Au substrate and Pt electrode as the reference. j–l) Reproduced with permission.^[^ ^149^ ^]^ Copyright 2016, Springer Nature.

Compared to the bulk materials, the nanostructured catalysts usually have a relatively large specific area, which will provide more active sites within the surface or at the edges and dramatically accelerate the catalytic process. One of the key advantages of 2D TMDs is their layered crystal structure, which shows intrinsic tendency to form atomic‐thickness NSs and sufficiently expose the catalytically active sites. It is considered that both the metal or nonmetal at the edge of TMDs NSs can serve as the catalytic sites,^[^ ^74^, ^82^, ^138^, ^139^ ^]^ and the most direct way to enhance the activity involves thinning the layers of TMDs, which could expose the embedded active sites.^[^ ^94^, ^140^ ^]^ Liquid exfoliation is a commonly used method to obtain the monolayer, or few‐layer NSs as introduced in Section 3.1. Nguyen et al. found the exfoliated MoS_2_ NSs had a much larger specific area and the layered structure would expose more active sites, resulting in an Tafel slope of 94.91 mV dec^−1^ and onset potential of about 100 mV (at 1 mA cm^−2^), whose catalytic activity showed obvious improvement compared to the bulk MoS_2_ (**Figure** **6**a–c).^[^ ^141^ ^]^ Moreover, different intercalation methods may also result in performance with various extent of improvement. Pumera et al. utilized different organolithium compounds (including *n*‐BuLi, Me‐Li, and *t*‐BuLi) to exfoliate the bulk MoS_2_ and found that larger organic compounds (such as *t*‐BuLi and *n*‐BuLi) could generate larger size anions, which was in favor of the intercalation process of Li^+^ and reduced the layer number of TMDs NSs.^[^ ^142^ ^]^ Thus, more active sites would be exposed after the intercalation process, and the HER performance would be significantly enhanced (Figure 6d–f). Briefly, by using the liquid exfoliation methods, the bulk TMDs could be thinned into single‐ or few‐layer NSs, and the active sites were sufficiently exposed, which would markedly improve the electrocatalytic performance.

The exfoliation strategy can efficiently improve the performance of the electrocatalysts toward HER; however, the exfoliating process usually lacks control, which may bring some disadvantages to the NSs, such as nonuniform size, inhomogeneous layer numbers and so on. This will hinder the investigation of the catalytic mechanism toward the TMDs.^[^ ^50^ ^]^ Compared to the chemical exfoliating strategy, the CVD method is a controllable route to prepare NSs with abundant fully exposed edges, which is considered to be a suitable model to investigate the catalytic mechanism. For instance, Liu et al. accomplished the growth of triangular single‐layer MoS_2_ flakes on Au substrate utilizing the low‐pressure CVD (LPCVD) technique (Figure 6g).^[^ ^114^ ^]^ Herein, about 80% area coverage of MoS_2_ on Au substrate demonstrated the best catalytic activity with a η of 300 mV (at 50.5 mA cm^−2^), which was ≈25 times over the bulk counterpart (Figure 6h). Moreover, increased edge length would also contribute to the reaction process by increasing the density of catalytic sites at edges (Figure 6i).

Creating defects is another route to improve the quantity of active sites of TMDs NSs on their basal plane. Generally speaking, the basal planes of the TMDs NSs are inert toward HER; however, introduction of moderate number of defects stimulates the catalytic activity of the basal plane.^[^ ^143^, ^144^, ^145^ ^]^ Various strategies such as plasma treatment,^[^ ^146^, ^147^ ^]^ chemical functionalization in liquid phase,^[^ ^20^, ^148^ ^]^ have been used to increase the defects density. Apart from increasing the active sites, appropriate concentration of defects would also tune the electronic structure. For instance, density functional theory (DFT) results indicated the increasing the S atom vacancies (S‐vacancies) could strengthen the hydrogen adsorption and strain the S‐vacancies sites, which would lead to optimal $\Delta {\text{G}}_{{\text{H}}^{\text{*}}}$ and make the gap states close to the Fermi level (*E*_F_) (Figure 6j,k).^[^ ^149^ ^]^ Thus, the HER activity could be effectively tuned up and the optimal MoS_2_ electrocatalysts were obtained (Figure 6l). Furthermore, theoretical investigation^[^ ^150^ ^]^ on the defects engineering for facilitating the HER performance also showed that when the defects were created on the basal surfaces of the TMDs, a dangling bond state closer to the *E*_F_ arisen, which activated the basal plane for HER.

Owing to the layered structure of TMDs‐based catalysts, we consider the methods that used to promote the density of active sites could be classified into two different types. One is augmenting the active sites at edges of TMDs NSs, either by reducing the layer number of the NSs, or by increasing the edges length. Nevertheless, the boundaries of the exfoliated TMDs NSs are usually irregular, which makes it very difficult to control the edges length. The other one is modulating the basal plane of the TMDs NSs. The vast majority of atoms in TMDs NSs exist within the basal plane, and there seems to be more potential to increase the active sites, if the defects exist in the basal plane rather than edges. Besides, the defects can also induce the variation of local electron density and subsequently modulated the electronic structure of TMDs NSs, which may further accelerate their HER process. Although creating defects can effectively influence the catalytic activity of TMDs NSs, its density must be controlled at a reasonable level. Insufficient defects may result in inconspicuous effect of the activity, but excess defects may serve as electron capture caterers, which in return hinder the transfer of electrons and make the catalysts poisoned.^[^ ^151^ ^]^

### 4.2 Heteroatoms Doping

Doping heteroatoms is a useful way that has been widely used to enhance the HER performance of TMDs electrocatalysts.^[^ ^152^ ^]^ Notably, doped heteroatoms will effectively modify electron structure especially the d‐band of TMDs‐based material and decrease the value of $\Delta {\text{G}}_{{\text{H}}^{\text{*}}}$ of the electrocatalysts, which facilitate the HER process. Both the metal sites^[^ ^132^, ^153^, ^154^, ^155^ ^]^ and nonmetal sites^[^ ^133^, ^156^, ^157^, ^158^, ^159^ ^]^ in TMDs can be partially replaced to improve the HER performance, which affords greater possibilities to modulate the basic properties of the host materials.

#### 4.2.1 Metal Doping

**Figure 7:**
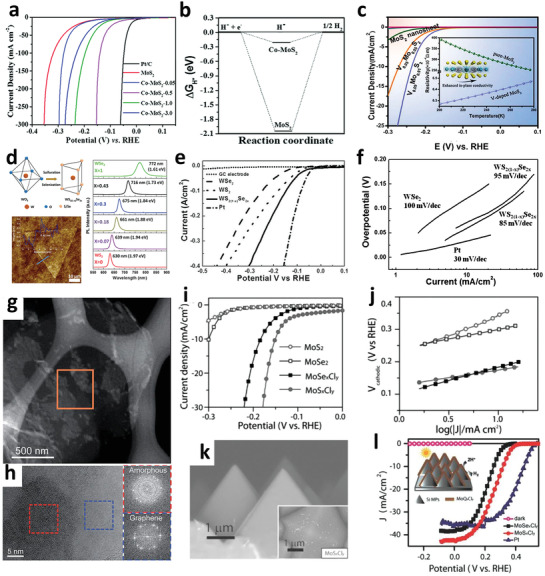
a) LSV curves of HER and b) the calculated $\Delta {\text{G}}_{{\text{H}}^{\text{*}}}$ of pure MoS_2_ and Co‐doped MoS_2_. a,b) Reproduced with permission.^[^ ^164^ ^]^ Copyright 2018, Royal Society of Chemistry. c) LSV curves of pure and V‐doped MoS_2_ NSs. Inset: demonstrated temperature‐dependent resistivity of V‐doped MoS_2_ NSs. Reproduced with permission.^[^ ^161^ ^]^ Copyright 2014, Royal Society of Chemistry. d) Preparation of hexagonal WS_2(1−_ *_x_*_)_Se_2_ *_x_* through simultaneous selenization and sulfurization reaction of monoclinic WO_3_. The room‐temperature PL spectra and the corresponding AFM image of the prepared WS_2(1−_ *_x_*_)_Se_2_ *_x_* NS. e) Comparison for HER catalytic activity for Pt, GC, monolayer WS_2_, WSe_2_, and WS_2(1−_ *_x_*_)_Se_2_ *_x_* (*x* = 0.43). f) The corresponding Tafel slope value. d–f) Reproduced with permission.^[^ ^157^ ^]^ Copyright 2015, Wiley‐VCH. g) EDS element mapping of a monolayer graphene NS that was partially covered by MoS*_x_* Cl*_y_*. h) HRTEM and local FFT image of a MoS*_x_* Cl*_y_*–VG sheet. g,h) Reproduced with permission.^[^ ^72^ ^]^ Copyright 2015, Royal Society of Chemistry. i) HER performance and j) their corresponding Tafel plot of MoS*_x_* Cl*_y_*, MoSe*_x_* Cl*_y_*, MoS_2_, and MoSe_2_. k) Cross‐sectional and top to down (inset) SEM images of MoS*_x_* Cl*_y_*/Si MPs. l) PEC‐HER performance of MoSe*_x_* Cl*_y_*/Si MPs (squares), MoS*_x_* Cl*_y_*/Si MPs (circles), and Pt/Si MPs (triangles) photocathodes. The experiments were conducted in 0.5 M H_2_SO_4_ under 1 Sun irradiation. i–l) Reproduced with permission.^[^ ^30^ ^]^ Copyright 2015, Wiley‐VCH.

Several metal elements (such as V, Co, Fe, Ni, Cu, and Zn) have already been used as the doping elements for the TMDs‐based electrocatalysts successfully. Generally, the doping can accelerate the HER process by increasing catalytic sites,^[^ ^92^, ^160^ ^]^ improving the conductivity,^[^ ^161^ ^]^ and/or the optimizing of electronic structure.^[^ ^132^ ^]^ However, the doping elements should be selected carefully because different dopants may bring different influence on HER activity of TMDs electrocatalysts. For example, Ni and Co doping were frequently‐used to improve the HER activity of MoS_2_.^[^ ^160^, ^162^, ^163^ ^]^ These two elements could reduce the value of $\Delta {\text{G}}_{{\text{H}}^{\text{*}}}$ and increase active sites density, which finally optimizing HER performance. Xiong et al. developed a one‐step hydrothermal method to prepare Co‐doped MoS_2_,^[^ ^164^ ^]^ which exhibited an enhancement of HER catalytic activity compared with pure MoS_2_ (**Figure 7**a). When the doping amount of Co source was 0.5 mmol, the sample Co‐MoS_2_‐0.5 demonstrated the best HER performance in alkaline media (η = 90 mV). Such improvement should be ascribed to the decreasing of $\Delta {\text{G}}_{{\text{H}}^{\text{*}}}$ (Figure 7b) and the regulated electronic structure, induced by Co doping. Different from the Co and Ni doping, Xie et al. demonstrated that doping with V atom would mainly improve the conductivity of MoS_2_ NSs, but not increase the density of active sites (Figure 7c).^[^ ^161^ ^]^

#### 4.2.2 Nonmetal Doping

Besides the metal doping, many types of nonmetal‐doped TMDs also show unique characteristics and excellent HER performance. Different from the metal doping strategy, nonmetal doping not only optimizes the $\Delta {\text{G}}_{{\text{H}}^{\text{*}}}$, but also results in crystal distortion or amorphous structure with plenty of active sites.^[^ ^133^, ^157^, ^165^ ^]^ Xie et al. prepared oxygen‐doped MoS_2_ NSs by hydrothermal method.^[^ ^166^ ^]^ Relatively low synthesis temperature led to MoS_2_ NSs inheriting a small quantity of Mo–O bonds. Further, DFT results indicated that MoS_2_ slab incorporated with oxygen showed a narrower bandgap of 1.30 eV, while the value of 2H MoS_2_ slab is 1.75 eV. This consequence of bandgap narrowing was caused by the oxygen incorporation into MoS_2_ NSs, which resulted in a higher carrier concentration and thus a better conductivity. As a result, optimized oxygen‐incorporated MoS_2_ catalyst exhibited a relatively high HER catalytic activity with a η of 120 mV (at 1 mA cm^−2^) and the Tafel slop of 55 mV dec^−1^. Fu et al. synthesized the Se doped WS_2(1−_ *_x_*_)_Se_2_ *_x_* single‐layer NSs and investigated the influence of Se doping.^[^ ^157^ ^]^ When the S atoms were superseded by a larger Se atom, the crystal distortion of the WS_2_ generated a polarized electric field, and the bond breaking of the H_2_O molecules was accelerated. Thus, single‐layer WS_2(1−_ *_x_*_)_Se_2_ *_x_* NS showed a relatively lower η of 80 mV to obtain 10 mA cm^−2^, while WSe_2_ and WS_2_ NSs needed 150 and 100 mV to obtain the same value, respectively (Figure 7d–f). Moreover, Jin’s group found that in case of both MoS_2_ and MoSe_2_ catalysts, doping with chlorine (Cl) resulted in effective improvement in their HER activity and tune the electronic structure of amorphous MoSe_2_ and MoS_2_ (Figure 7g,h).^[^ ^30^, ^72^ ^]^ The combination of the optimal electronic structure and the numerous active sites finally enhanced the HER performance of MoS*_x_* Cl*_y_* and MoSe*_x_* Cl*_y_* (Figure 7i,j).^[^ ^30^ ^]^ Moreover, amorphous MoQ*_x_* Cl*_y_* (Q = S, Se) was grown on the n^+^pp^+^ Si substrate with micropyramids (MPs), constituting a highly efficient PEC‐HER photocathodes (Figure 7k). These PEC‐HER photocathodes demonstrated a HER activity of 43 and 38.8 mA cm^−2^ at 0 V versus reversible hydrogen electrode (RHE) for MoS*_x_* Cl*_y_*/Si and MoS*_x_* Cl*_y_*/Si, respectively. These values were even better than that of the Pt/Si photocathodes (Figure 7l).

Generally, doping with heteroatoms can improve the HER performance of TMDs catalysts in two different aspects. First, the element doping can induce crystal distortion and make the density of catalytic sites increasing. Second, due to different electron configuration, the dopants can significantly modulate the electronic structure of the catalysts, and this may lead to the optimizing of $\Delta {\text{G}}_{{\text{H}}^{\text{*}}}$ or the matching degree of energy level. Nevertheless, the dopants still need to be carefully selected, since different atoms would have various effect on catalytic activity and improper elements may even have a negative influence on the final performance. Another confusing result is that, even doping with the same elements, the HER catalytic activities may show various results, sometimes even inversed.^[^ ^153^, ^160^ ^]^ In our opinion, this may be caused by different preparation methods and doping amount, which results in the dopants anchoring on different sites of the TMDs NSs. But the inner mechanism still needs further theoretical and experimental investigation.

### 4.3 Phase Engineering

**Figure 8:**
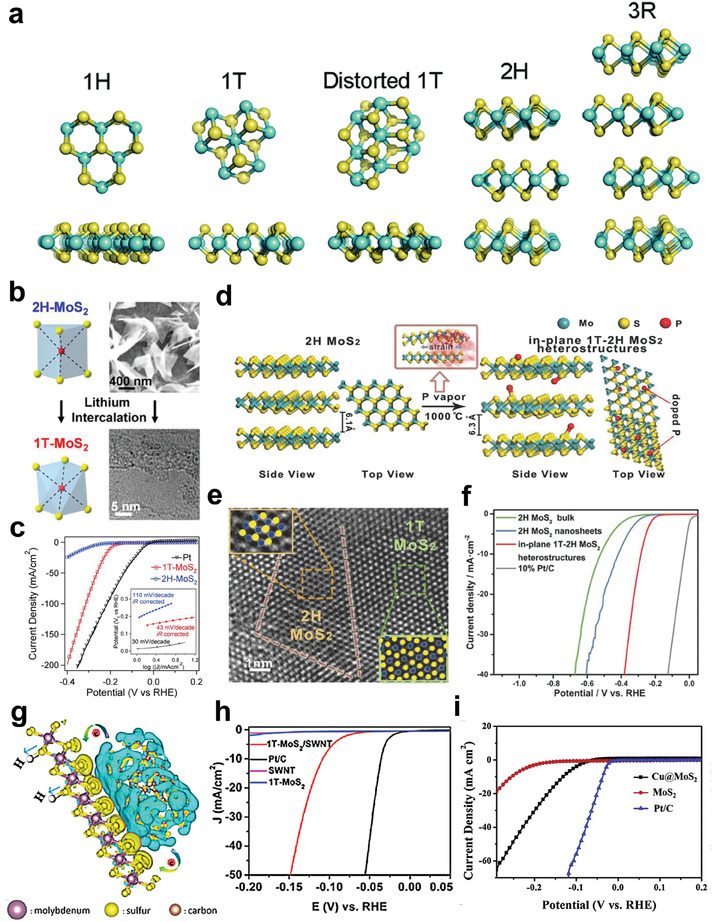
a) Different crystal structures of TMDs, from left to right: 1H phase, ideal 1T phase, 1T′ phase (which is actually the distorted 1T phase), 2H phase, and 3R phase. Reproduced with permission.^[^ ^167^ ^]^ Copyright 2015, Royal Society of Chemistry. b) Lithium‐intercalation‐triggered phase transform of MoS_2_ (2H to 1T phase), and c) comparison of HER performance for the two different electrocatalysts. b,c) Reproduced with permission.^[^ ^85^ ^]^ Copyright 2013, American Chemical Society. d) Preparation process of the partial transformed MoS_2_ NSs, which was induced by doping with phosphorus atoms. e) HRTEM image of in‐plane heterostructures in MoS_2_ NSs with f) LSV curves for MoS_2_ with in‐plane 1T‐2H heterostructures, 2H MoS_2_ bulk, 2H MoS_2_ NSs, and 10% Pt/C. d–f) Reproduced with permission.^[^ ^175^ ^]^ Copyright 2018, Wiley‐VCH. g) Calculated charge density distribution around the 1T MoS_2_ and SWNT interface. The yellow area represents the electrons gathering, while the blue area represents electrons losing area, respectively. h) HER performance of commercial Pt catalyst, 1T MoS_2_/SWNT heterostructure, SWNT, and 1T MoS_2_. g,h) Reproduced with permission.^[^ ^176^ ^]^ Copyright 2017, American Chemical Society. i) LSV curves of commercial Pt/C, MoS_2_ and single Cu atom doped MoS_2_. Reproduced with permission.^[^ ^177^ ^]^ Copyright 2019, Elsevier.

Most TMDs with layered structure have the similar chemical formula which can written as MX_2_ (M represents Mo, W, Nb, and other transition metal, while X is S, Se, or Te). One unique feature of TMDs electrocatalysts is the existence of multiple crystal phases. **Figure 8**a exhibits five different crystal structures observed in MoS_2_. Among them, 1H, 2H, and 3R phases correspond to semiconductor (2H are usually used as the raw materials for catalysts preparation), while the 1T and 1T′ (distorted 1T) are metallic phases.^[^ ^167^, ^168^ ^]^ Owing to their larger density of the active sites, metallic conductivity, and better electrode kinetics, 1T and 1T′ TMDs are usually considered to be more favorable for HER compared to other phases, and significant research efforts have been devoted to converting 2H phase into 1T/1T′ phase.^[^ ^151^, ^169^, ^170^, ^171^, ^172^, ^173^ ^]^ Jin’s group found that the MoS_2_ with metallic 1T phase, obtained by exfoliating the 2H MoS_2_ by *n*‐BuLi (Figure 8b),^[^ ^85^ ^]^ exhibited a much better HER performance compared with the 2H phase MoS_2_ (Figure 8c). The activity enhancement was induced by the increased active sites number of MoS_2_ NSs at their edges and the metallic property of 1T polymorph. From then on, several studies have concentrated on chemical exfoliating the bulk TMDs by lithium intercalation to obtain single‐ or few‐layered 1T/1T′ MX_2_ NSs with excellent HER activities.^[^ ^33^, ^84^, ^87^, ^174^ ^]^ Besides exfoliation, element doping will also induce phase transition of 2H TMDs NSs. For instance, if annealing the 2H MoS_2_ in phosphorus vapor, the crystal phase could be partially converted from 2H to 1T and form the in‐plane 1T‐2H MoS_2_ heterostructures (Figure 8d,e).^[^ ^175^ ^]^ The doped phosphorus atoms would not only be embedded into the lattice of MoS_2_, which induced the phase transition, but also further promote the conductivity. The improved electrical conductivity, more catalytic sites, and hydrophilic property enabled the in‐plane 1T‐2H MoS_2_ heterostructures acquire an ultrastable and relatively high HER performance in alkaline media (Figure 8f). Although phase transition from 2H to 1T/1T′ have greatly promoted the HER activity of TMDs, there is still room to further improve their catalytic performance. Recently, Liu et al.^[^ ^176^ ^]^ proposed an electron doping method by in situ forming 1T MoS_2_/ single‐walled carbon nanotube (SWNT) heterostructure. Theoretical simulation results showed that after the formation of heterostructure, electrons would tend to migrate from the SWNT to 1T MoS_2_ and accumulate around the S atoms in 1T MoS_2_ (Figure 8g). The injected electrons would not only weaken the adsorption capacity of H atoms on the catalyst’s surface, but also accelerate H atom recombination and H_2_ release processes, which finally enhanced the HER performance of 1T MoS_2_ (Figure 8h). Apart from constructing heterostructure, single atom Cu integrated on 1T MoS_2_ could also exhibit a similar effect (Figure 8i).^[^ ^177^ ^]^

It is noteworthy that the 2H TMDs could not be completely transformed into 1T phase. The percentage of 1T phase was usually around 80%^[^ ^20^, ^178^ ^]^ or less,^[^ ^27^, ^179^, ^180^ ^]^ which indicated that the obtained electrocatalysts were the mixed phases of 1T and 2H. Moreover, many 1T TMDs, such as 1T MoS_2_, 1T WS_2_, could be easily converted back into the more stable 2H phases, which resulted in the decrease of HER performance.^[^ ^168^, ^170^, ^181^ ^]^ The shortcoming is particularly obvious when it comes to the preparation of wafer scale 1T/1T′ TMDs films on various substrates, which could be used for the fabrication of devices or catalytic mechanism investigation. For now, the preparation pathways of TMDs film in wafer scale are very limited, mainly including CVD, PLD and MBE etc. Since the preparation process are often conducted under high temperature, the final 2H phase products are usually obtained. Even if the 1T/1T′ phases are successfully obtained, it will be hard to guarantee the uniformity of the large size film and its catalytic activities still needed to be further improved. So, increasing the phase conversion ratio from 2H to 1T/1T′ and improving stability of final products are two main obstacles that should be overcome and more focus should be paid on the investigation of optimal synthetic strategies.

### 4.4 Construction of Heterostructures

A variety of heterostructured materials have been proposed to be excellent electrocatalysts for HER recently.^[^ ^131^, ^182^, ^183^, ^184^, ^185^, ^186^, ^187^, ^188^ ^]^ Compared to the single species of active substance, catalysts with composite structures show some distinct advantages.

#### 4.4.1 Increase of the Electronic Conductivity

**Figure 9:**
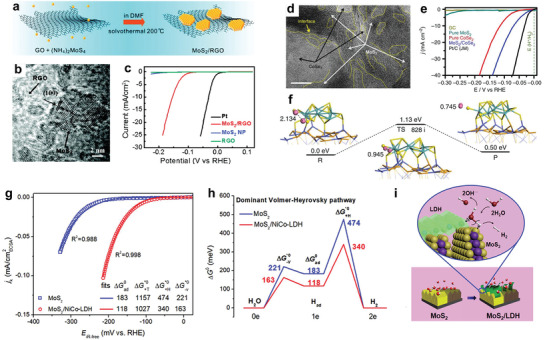
a) Synthetic route of the composite MoS_2_/RGO electrocatalyst. b) HRTEM of the as‐synthesized MoS_2_/RGO hybrid catalysts. c) LSV curves of MoS_2_, RGO, MoS_2_/RGO hybrid and Pt catalyst. a–c) Reproduced with permission.^[^ ^197^ ^]^ Copyright 2011, American Chemical Society. d) HRTEM of MoS_2_/CoSe_2_ after stability test, wherein more MoS_2_‐CoSe_2_ interfaces were exposed. Scale bar: 10 nm. e) HER activity of pure MoS_2_, pure CoSe_2_, bare GC electrode, GC electrodes modified with MoS_2_/CoSe_2_ hybrid, and the Pt/C catalyst. f) Pathway and calculated energy barrier for HER process on the surface of MoS_2_/CoSe_2_ hybrid according to the Volmer–Tafel mechanism. The pink, azure, blue, yellow, and orange balls indicate the H Mo, Co, S, and Se, respectively. d–f) Reproduced with permission.^[^ ^105^ ^]^ Copyright 2015, Springer Nature. g) LSV curves normalized to the ECSA for MoS_2_/NiCo‐LDH and bare MoS_2_. h) Free energy of HER in alkaline solution for MoS_2_/NiCo‐LDH and bare MoS_2_, which went through the Volmer–Heyrovsky mechanism. i) The diagram of HER progress at heterojunction interfaces of MoS_2_/NiCo‐LDH and bare MoS_2_ in alkaline media. g–i) Reproduced with permission.^[^ ^209^ ^]^ Copyright 2017, Cell Press.

Coupling TMDs electrocatalysts with conductive species, such as graphene,^[^ ^45^, ^189^, ^190^, ^191^ ^]^ carbon paper,^[^ ^192^, ^193^ ^]^ and metal substrate^[^ ^194^, ^195^, ^196^ ^]^ is a commonly used method to promote their HER activities. Integration of the TMDs‐based electrocatalysts and the conductive substrates not only provides a conductive framework with numerous internal electron transport channels, but also increases the effective ECSA and active sites for HER process. For example, in 2011, Dai’s group synthesized a composite electrocatalyst with reduced graphene oxide (RGO) and MoS_2_ NSs via a convenient solvothermal technique (**Figure** **9**a,b).^[^ ^197^ ^]^ Composite MoS_2_/RGO material demonstrated a better HER activity than the simple MoS_2_ particles, which showed a η of 100 mV (Figure 9c). In this case, the HER performance enhancement was attributed to adequately exposed active sites at edges of MoS_2_.Also, the coupling with the graphene network would accelerate the carrier migration efficiency from the MoS_2_ to the electrodes. Compared to the combination of TMDs and other carbon materials (e.g., RGO, carbon nanotubes, carbon nanofibers), 3D conductive metal substrates (such as Cu foam, Ni foam, Fe foam, and Ti foam) can provide a relatively large surface area, beneficial for significant increase in the loading amount of catalysts.^[^ ^198^, ^199^, ^200^ ^]^ Notably, coupling with conductive scaffolds can only optimizing the catalytic activity by increasing the catalytic surface area and promote the electrons transfer between the substrates and the catalysts; nonetheless, the intrinsic activity of the catalyst usually remains unchanged.^[^ ^19^, ^201^ ^]^

#### 4.4.2 Optimization of Kinetic Process

One of the important roles of heterostructure involves the acceleration of the directional migration of the electrons with properly matched band structures in the different combined materials, from the perspective of semiconductor physics.^[^ ^69^ ^]^ HER is a surface electrochemical process which depends highly on the effective interaction between the catalyst and hydrogen in the electrolyte.^[^ ^202^, ^203^ ^]^ As mentioned in Section 2.2.1, $\Delta {\text{G}}_{{\text{H}}^{\text{*}}}$ is an important descriptor to evaluate the utilization potential of a HER catalyst. Construction of the heterostructured catalysts results in effective optimization of the reaction kinetic process. Yu et al. fabricated the MoS_2_/CoSe_2_ heterostructure via a simple hydrothermal method (Figure 9d clearly exhibits the heterostructure of MoS_2_ and CoSe_2_).^[^ ^105^ ^]^ The as‐prepared hybrid electrocatalyst needed a η of 75 mV (to reach 10 mA cm^−2^) and Tafel slope was 36 mV dec^−1^. Both of them were lower than the MoS_2_ and CoSe_2_ and even analogous to benchmark Pt/C electrocatalyst (Figure 9e). In this study, the heterostructure of MoS_2_/CoSe_2_ plays two important roles for enhancing the catalytic activity. On one hand, the formation of MoS_2_ and CoSe_2_ interfaces would bring in lots of interfaces with rich catalytic sites. On the other hand, the heterostructure can obviously optimize $\Delta {\text{G}}_{{\text{H}}^{\text{*}}}$ (Figure 9f) and make the value close to Pt (111).^[^ ^204^ ^]^ The moderate $\Delta {\text{G}}_{{\text{H}}^{\text{*}}}$ could effectively reduce the reaction barrier, and H atoms could form H_2_ molecules more facilely. Moreover, the adsorption energy of the final H_2_ molecules was as low as 0.5 eV, which would be conducive to the release of H_2_ from the active sites, and further accelerate the HER process. In industry, chlor‐alkali electrolysis is a widely used technique to produce hydrogen.^[^ ^52^, ^205^ ^]^ However, the alkaline HER process is usually more sluggish compared to that in acid media, which is largely limited by the slow kinetics of water dissociation.^[^ ^206^ ^]^ So, it is an important task to explore high‐efficient alkaline HER catalysts for the purpose of reducing the energy consumption in chlor‐alkali industry. Owing to the lack of H^+^ in alkaline electrolyte, the HER process initiates from water dissociation, which requires additional energy to generate protons. Construction of heterostructured catalysts with multiple functional sites is an efficient approach to accelerate the overall process. For example, Yang and the coauthors synthesized hierarchical composite with MoS_2_ and NiCo layered double hydroxide (MoS_2_/NiCo‐LDH), which exhibited an enhanced HER catalytic activities in alkaline media than bare MoS_2_ (Figure 9g). DFT calculations results also demonstrated that the activation energies for Heyrovsky and Volmer steps of MoS_2_/NiCo‐LDH were both lower than bare MoS_2_ (Figure 9h). The MoS_2_ at the interface favored the chemisorption of H, while the LDH could effectively adsorb the hydroxyl species.^[^ ^207^, ^208^ ^]^ Therefore, water molecules at the hetero‐interface could be more easily dissociated, in favor of the rate‐determining Volmer step as shown in Figure 9i and thus promoted the HER catalytic process in alkaline media.^[^ ^209^ ^]^ Similar results have also been obtained in MoS_2_/NiFe‐LDH,^[^ ^210^ ^]^ Ni(OH)_2_/MoS_2_,^[^ ^183^ ^]^ MoS_2_/Ni_2_S_3_,^[^ ^211^ ^]^ and NiS_2_/MoS_2_,^[^ ^212^ ^]^ in which the TMDs serve as the catalytic species, while the other ingredient helps accelerating the water dissociation rate.

### 4.5 Synergistic Modulation

**Figure 10:**
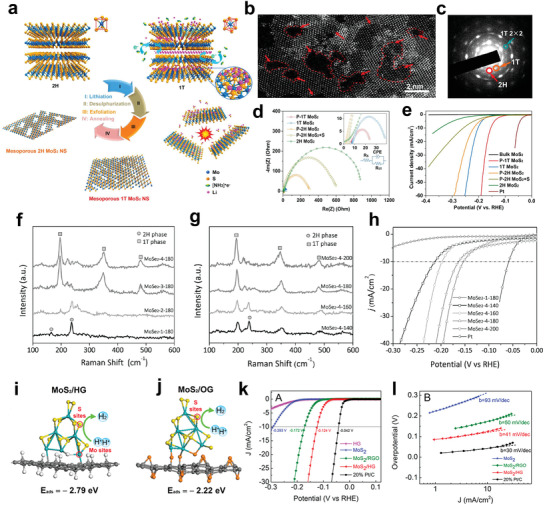
a) Diagram for the preparation process of 2H MoS_2_, 1T MoS_2_, P‐2H MoS_2_, P‐1T MoS_2_, and P‐2H MoS_2_+S. b) HR‐STEM and c) SAED pattern of P‐1T MoS_2_ NSs. d) The electrochemical impedance spectroscopy Nyquist plots and e) HER performance of various MoS_2_ samples. a–e) Reproduced with permission.^[^ ^20^ ^]^ Copyright 2016, American Chemical Society. f) The comparison of Raman spectra for MoSe_2_ NSs prepared with different amounts of NaBH_4_ at 180 °C. g) Raman spectra of the MoSe_2_ NSs synthesized under different temperatures with the same amount of NaBH_4_. h) HER activities of various MoSe_2_ samples synergistically modified by phase and disorder engineer. f–h) Reproduced with permission.^[^ ^27^ ^]^ Copyright 2018, Wiley‐VCH. i–j) The DFT calculation result of hydrogen adsorption energy at S edge sites of MoS_2_ on HG and OG. k) LSV curve of different MoS_2_ electrocatalysts and l) their Tafel slops. i–l) Reproduced with permission.^[^ ^216^ ^]^ Copyright 2018, American Chemical Society.

All the strategies mentioned above could benefit the HER process; however, to promote the HER performance of the TMDs materials to the greatest extent, two or more factors should be synergistically regulated. For example, the exfoliation of bulk MX_2_ by lithium intercalation method will significantly increase the active sites number, and at the same time, the exfoliation can lead to partial phase conversion of MX_2_ based catalysts from inert 2H phase to highly active 1T phase. Thus, after the lithium intercalation, both the active sites number and electrical conductivity can get increased, which synergistically optimizes the HER activities.^[^ ^43^, ^87^, ^213^, ^214^ ^]^ Our previous work demonstrated that by synergistically modulating the edges, S‐vacancies, and crystal structure (phase), the HER performance of MoS_2_ could be remarkably improved.^[^ ^20^ ^]^ Systematic investigation of the HER performance of a series representative MoS_2_ NSs, including 2H MoS_2_, 1T MoS_2_, porous 2H MoS_2_ (P‐2H MoS_2_), porous 1T MoS_2_ (P‐1T MoS_2_), and porous 2H MoS_2_ with compensated S(P‐2H MoS_2_+S) (**Figure 10**a), indicated exfoliated porous MoS_2_ NSs could provide more catalytic active sites by introducing more S‐vacancies and edges into the basal plane of MoS_2_ NSs (Figure 10b). However, the phase transition could not only enhance conductivity, but also activate the inert basal planes of MoS_2_ NSs (Figure 10c,d), which was considered to be the crucial factor to determine the HER performance. Our work clearly implies that synergistic modulation of multiple factors will bring in a better HER activity rather than single modulation aspect (Figure 10e).

Similar to the modulation of MoS_2_, HER performance for MoSe_2_ could also be enhanced with regulating density of active sites and intrinsic activity synergistically. Thus a modified one‐pot hydrothermal method was proposed to synthesize highly active MoSe_2_ NSs by utilizing phase and disorder engineering together.^[^ ^27^, ^215^ ^]^ With excess amount of NaBH_4_, a severe reduction process resulted in the crystal phase of MoSe_2_ partially converting from 2H to 1T (Figure 10f), which largely improved in the conductivity and intrinsic activity of the MoSe_2_ NSs. Also, the lower reaction temperature could increase the degree of disorder in the MoSe_2_ NSs and provided abundant unsaturated defects serving as the active sites. However, low temperature was not favorable for the formation of 1T MoSe_2_, and when the temperature was below 180 °C, the 2H phase became distinct (Figure 10g), which was unfavorable for the HER performance of MoSe_2_. Consequently, by simultaneously modulating reaction temperature and the ratio of NaBH_4_, the best optimized MoSe_2_ sample was obtained when the molar ratio of NaBH_4_ to Mo source was 4 and reaction temperature was 180 °C. The η for the best sample was 152 mV (at 10 mA cm^−2^), and the Tafel slope for the sample was 52 mV dec^−1^ (Figure 10h).

Synergistic modulation means multitudinous combining form of different strategies. As for TMDs‐based electrocatalysts, their layered structure, unique physical and chemical properties, different crystal phases, and various preparation methods, can all be modulated, which give the final products unlimited possibilities. In fact, almost all methods mentioned from Section 4.1 to 4.4 will involve two or more modulating aspects. For example, the combination of TMDs‐based catalysts with conductive substrate would improve the conductivity of the catalysts and simultaneously create more catalytic sites around the interfaces.^[^ ^216^ ^]^ Simultaneously, proper heterostructure would optimize the $\Delta {\text{G}}_{{\text{H}}^{\text{*}}}$ and reduce the reaction barrier to facilitate HER (Figure 10i–l). Similarly, doping with heteroatoms may simultaneously increase of active sites and induce the phase transition, besides the tuning of electronic structure.^[^ ^161^, ^176^ ^]^ It enlightens us that, as a complex surface electrochemical process, single modulation strategy may change more than one property of the electrocatalysts, and only comprehensive analysis of all possible influence factors, the optimal method could be obtained.

## 5 Summary and Outlook

Herein, some important advances of 2D TMDs electrocatalysts are systematically summarized. First, two important HER mechanisms and some key descriptors for evaluating the HER activity are introduced to give readers a basic understanding of this cathodic reaction. Subsequently, various methods for synthesizing the TMDs electrocatalysts are reviewed. Exfoliation from bulk materials could directly result in the preparation of few or single layer TMDs, wherein more active sites get exposed. The review also presents that the TMDs nanomaterials with different morphological characteristics, phases, or crystallinity could be conveniently prepared by hydrothermal or solvothermal method, during which the performance of the catalysts could be tuned from multiple aspects. CVD is considered to be an ideal way for the preparation of low‐defect, high‐quality, and layer controllable nanostructures on various substrates to investigate the HER catalytic mechanism of 2D TMDs. Other methods such as CVT, MBE are also commonly utilized methods to prepare high‐quality 2D TMDs nanomaterials. Finally, various approaches that usually used to accelerate the HER performance of TMDs are mainly introduced. In general, to enhance the HER activities of certain catalysts, two basic aspects should be comprehensively considered: increasing the active sites number and promoting the performance of each site. Exfoliation of bulk TMDs materials and generation of defects on the basal plane are two efficient approaches for increasing the amount of active catalytical sites either within basal plane or at the edge of the TMDs NSs. Heteroatoms doping and phase transition (usually generating more metallic 1T phase) can improve the conductivity and optimize electronic structure of TMDs‐based catalysts, which promote the intrinsic catalytic activity. Besides, construction of heterostructures is also an efficient way to modulate the kinetic process of HER (such as improvement in the water dissociating process) and facilitate the transportation of electrons from the electrocatalysts to electrolyte. Synergistic application of more than one approach mentioned above may result in better catalytic activity toward HER compared to that by following one single way, which is also called the “synergistic effect.”^[^ ^217^ ^]^ For a direct comparative overview, the HER performances of representative TMDs‐based electrocatalysts and the applied modulating strategies that were introduced herein are listed in **Table** **1**.

**Table 1:** Catalytic activity comparison for different TMDs‐based HER electrocatalysts

Catalyst	Electrolyte	*j* [mA cm^−2^]	η [mV]	Tafel slope [mV dec^−1^]	Main modulation strategies	Ref.
MoS_2_ NSs	0.5 m H_2_SO_4_	1	≈100	94.91	Liquid exfoliation	[141]
MoS_2_ NSs	0.5 m H_2_SO_4_	1	≈230	96	Liquid exfoliation	[142]
MoS_2_ flakes/Au foil	0.5 m H_2_SO_4_	50.5	300	61	Increasing edges active sites	[114]
SV‐MoS_2_	pH = 0.2	10	170	60	Vacancies engineering	[149]
Co‐MoS_2_/BCCF‐21	1 m KOH	10	48	52	Metal doping	[132]
Co‐MoS_2_‐0.5	1 m KOH	10	90	50.28	Metal doping	[164]
V_0.09_Mo_0.91_S_2_	1 m H_2_SO_4_	1	130	69	Metal doping	[161]
MoS*_x_* Cl	0.5 m H_2_SO_4_	10	150	48	Nonmetal doping	[30]
MoSe*_x_* Cl	0.5 m H_2_SO_4_	10	183	82	Nometal doping	[30]
WS_1.14_Se_0.86_	0.5 m H_2_SO_4_	10	80	85	Nonmetal doping	[157]
1T MoS_2_	0.5 m H_2_SO_4_	10	187	43	Phase engineering	[85]
1T’ MoS_2_	0.5 m H_2_SO_4_	10	175	100	Phase engineering	[170]
2H‐1T MoS_2_	0.5 m H_2_SO	10	136	73	Phase engineering	[151]
1T‐2H MoS_2_	1 m KOH	20	320	65	Phase engineering	[175]
1T MoS_2_/SWNT	0.5 m H_2_SO	10	108	36	Electron doping	[176]
Cu@MoS_2_	0.5 m H_2_SO	10	131	51	Electron doping	[177]
MoS_2_/CoSe_2_	0.5 m H_2_SO	10	75	36	Constructing heterostructure	[105]
Ni(OH)_2_/MoS_2_	1 m KOH	10	80	60	Constructing heterostructure	[183]
MoS_2_/RGO	0.5 m H_2_SO	1	≈100	41	Constructing heterostructure	[197]
MoS_2_/NiCo‐LDH	1 m KOH	10	78	76.6	Constructing heterostructure	[209]
MoS_2_/NiFe‐LDH	1 m KOH	10	110	77	Constructing heterostructure	[210]
MoS_2_/Ni_2_S_3_	1 m KOH	10	≈110	83.1	Constructing heterostructure	[211]
NiS_2_/MoS_2_	1 m KOH	10	204	65	Constructing heterostructure	[212]
P‐1T MoS_2_	0.5 m H_2_SO	10	154	43	Phase and defect engineering	[20]
1T MoSe_2_	0.5 m H_2_SO	10	152	52	Phase and defect engineering	[27]
MoS_2_/HG	0.5 m H_2_SO	10	124	41	Electronic structure modulation and interface engineering	[216]
2H‐MoS_0.2_Se_1.8_	0.5 m H_2_SO	10	136	50	Nonmetal doping and defect engineering	[133]

Although significant achievements have been made in this field, and plentiful electrocatalysts based on the TMDs materials have been designed and prepared, some challenges still hinder the commercial application for sustainable hydrogen production. Also, a lot more systematic explorations are needed in this field.

### 5.1 Mechanism Investigation

Despite the advances in TMDs‐based HER catalysts, the catalytic mechanism for most of them still lacks comprehensive understanding. It is thus important to deeply investigate the underlying mechanism, for offering rational guidance to design future HER catalysts. Burgeoning in‐situ/operando characterization methods, such as operando Raman,^[^ ^218^ ^]^ operando X‐ray diffraction (XRD),^[^ ^219^ ^]^ operando X‐ray absorption near‐edge structure (XANES),^[^ ^220^ ^]^ can help us deeply understand the real catalytic process and the composition‐performance relationship of the TMDs‐based electrocatalyst. The in‐depth investigation of the catalytic mechanism in return offers rational guidance to develop new TMDs‐based HER catalysts.

### 5.2 Combination of Theoretical and Experimental Studies

Recently, DFT calculations have attracted significant research attention and been extensively used in the field of electrochemical water splitting.^[^ ^221^, ^222^, ^223^, ^224^, ^225^ ^]^ Use of DFT offers a powerful computational tool to uncover the invisible electrochemical process, which aids in providing more fundamental insights into the real catalytic courses. Moreover, theoretical calculation is a powerful methods to screen possible catalyst candidates for HER^[^ ^226^, ^227^, ^228^ ^]^ and many of them have also been successfully prepared and utilized.^[^ ^1^, ^74^, ^138^ ^]^ Knowledge gained from the DFT results can be utilized to guide the materials designing, and the experimental results can further stimulate the additional theoretical researches. The virtuous feedback loop between the theoretical and experimental studies can significantly accelerate the new discoveries and inspires researchers to discover versatile ideas governing the fundamental phenomenon related to 2D TMDs.

### 5.3 Coupling with External Driving Field

Currently, modulation strategies of TMDs‐based electrocatalysts mainly focus on optimizing the properties of the catalysts themselves. However, with the rapid development of various TMDs‐based catalysts, there seems to be a bottleneck for catalytic activities enhancement and mechanism innovation. To further improve the catalytic effect, applying external driving fields during the test process may be a potential choice. According to some recent works, electric field^[^ ^229^, ^230^ ^]^ and magnetic field^[^ ^231^, ^232^ ^]^ could significantly improve the water splitting efficiency by tuning either the conductance or the spin polarization of the catalysts. It is speculated that HER performance of such novel catalysts could be further promoted with the assistance of the external driving field.

### 5.4 Designing New Type of TMDs‐Based Electrocatalysts

TMDs‐based materials have shown virtually unlimited potential for the HER, considering their various unique and fascinating chemical and physical properties. However, only few of the TMDs‐based layered catalysts, such as MoS_2_ (the most studied TMDs electrocatalyst), MoSe_2_, and WS_2_ have been well investigated so far. In fact, layer‐structured TMDs constitute a large family and many other members are still unexplored. To enrich our understanding of the 2D TMDs, other related materials should also be investigated. For example, TaS_2_,^[^ ^146^, ^233^ ^]^ NbS_2_,^[^ ^136^, ^234^ ^]^ and VS_2_,^[^ ^235^, ^236^ ^]^ all show their potential for electrochemical hydrogen generation. Therefore, searching for new types of TMDs‐based materials with favorable characteristics such as cost effectiveness, high efficiency, good durability, and pH‐independence, is essential for the future industrial water splitting application.^[^ ^59^ ^]^ Besides searching for single‐phase TMDs electrocatalysts, constructing proper heterostructure is another efficient strategy to optimize the electrochemical properties of the TMDs related materials.^[^ ^25^, ^56^, ^186^ ^]^ However, the integration of two different materials usually relies on the close contact and the formation of chemical bonds, which requires the similar lattice constant and limits the choices of raw materials.^[^ ^237^ ^]^ Recently, novel van der Waals (vdW) heterostructures have attracted lots of attention in many fields.^[^ ^238^, ^239^, ^240^ ^]^ These kinds of heterostructures are physically assembled together with different 2D materials by weak vdW interactions, which does not depend on chemical bonds. The simple integration of different 2D materials will inherit their respective properties and functionalities, which would vastly expand the application of layered materials. Some recent works have demonstrated that the combination of 2D TMDs and other layered materials would significantly improve the HER activities, especially in alkaline media.^[^ ^210^, ^241^, ^242^ ^]^ Nevertheless, the current strategies that used to construct vdW heterostructures usually involves in liquid or CVD methods, which makes the physicochemical characters of the products uncontrollable, such as layer number, hierarchical porosity and homogeneity. Therefore, versatile and facile techniques should be further investigated for controllable construction of vdW heterostructures.

### 5.5 Developing Standardized Testing

Due to the various testing conditions, it is very difficult to directly compare HER activities of electrocatalysts obtained by disparate researchers. Even for the same material, the different activities were found.^[^ ^141^, ^142^ ^]^ For example, most current densities reported are normalized by geometrical area of electrodes, in which the real effective catalytic area and loading amount of electrocatalysts are ignored, and this will have an obvious influence on the judgment of the catalytic activity. Hence, to comprehensively estimate the HER performance and benefit the comparisons of different TMDs‐based catalysts, standardized testing process should be applied. Some crucial tests, including catalyst loading, applied substrate, and the current densities, which are normalized to both electrochemical surface area and geometrical area, must be conducted.^[^ ^63^, ^168^ ^]^

### 5.6 Developing Integrated PEC‐HER Systems

PEC hydrogen generation is an effective method to utilize solar energy.^[^ ^243^, ^244^ ^]^ With the rapid expansion of high‐efficient TMDs‐based electrocatalysts, more efforts should be paid on PEC system. Two main factors should be considered to build an effective PEC‐HER photocathode: 1) The light absorption layer. Nowadays, silicon is the most used semiconductor for photocathode, but the stability of the Si‐based photo cathodes is not satisfying, especially in the alkaline media. Therefore, proper protect layer (such as Al_2_O_3_, TiO_2_, and Ni) should be coated on Si substrate to avoid Si dissolving in alkaline media.^[^ ^245^, ^246^ ^]^. 2) The proper catalysts. A well‐designed PEC‐HER system needs a proper catalyst that demonstrated high HER performance, as well as good optical transparency to ensure the sunlight reach the absorption layer. TMDs‐based electrocatalyst are promising choice for the catalytic layer, but the coating usually involves high temperature process to make the close contact with the light adsorption layer, which makes the 1T/1T′‐TMDs very difficult to serve as the catalyst. Jin’s group has done a pioneering work to coating 1T MoS_2_ on Si substrate via CVD methods,^[^ ^246^ ^]^ and this will give us an enlightenment to explore more efficient way to prepare TMDs‐based photocathodes.

In summary, the combination of theoretical and experimental study has demonstrated its great potential to investigate the catalytic mechanism. Under the guidance of theoretical simulations, various TMDs‐based electrocatalysts and novel modulation strategies should be further developed. At the same time, standardized testing could benefit the comparation of catalytic activities obtained from different groups. With all these efforts, we can speculate that a further development in TMDs‐based materials can be achieved in the field of water electrolysis.

## Conflict of Interest

The authors declare no conflict of interest.
